# ACA-28, an ERK MAPK Signaling Modulator, Exerts Anticancer Activity through ROS Induction in Melanoma and Pancreatic Cancer Cells

**DOI:** 10.1155/2024/7683793

**Published:** 2024-03-11

**Authors:** Teruaki Takasaki, Yasuyuki Hamabe, Kenta Touchi, Golam Iftakhar Khandakar, Takeshi Ueda, Hitoshi Okada, Kazuko Sakai, Kazuto Nishio, Genzoh Tanabe, Reiko Sugiura

**Affiliations:** ^1^Laboratory of Molecular Pharmacogenomics, Department of Pharmaceutical Sciences, Faculty of Pharmacy, Kindai University, Osaka 577-8502, Japan; ^2^Department of Biochemistry, Faculty of Medicine, Kindai University, Osaka 589-8511, Japan; ^3^Anti-Aging Center, Kindai University, Osaka 577-8502, Japan; ^4^Department of Genome Biology, Faculty of Medicine, Kindai University, Osaka 589-8511, Japan; ^5^Laboratory of Organic Chemistry, Department of Pharmacy, Faculty of Pharmacy, Kindai University, Osaka 577-8502, Japan

## Abstract

The extracellular signal-regulated kinase (ERK) MAPK pathway is dysregulated in various human cancers and is considered an attractive therapeutic target for cancer. Therefore, several inhibitors of this pathway are being developed, and some are already used in the clinic. We have previously identified an anticancer compound, ACA-28, with a unique property to preferentially induce ERK-dependent apoptosis in melanoma cells. To comprehensively understand the biological cellular impact induced by ACA-28, we performed a global gene expression analysis of human melanoma SK-MEL-28 cells exposed to ACA-28 using a DNA microarray. The transcriptome analysis identified nuclear factor erythroid 2-related factor 2 (Nrf2), a master transcription factor that combats oxidative stress, as the most upregulated genetic pathway after ACA-28 treatment. Consistently, ACA-28 showed properties to increase the levels of reactive oxygen species (ROS) as well as Nrf2 protein, which is normally repressed by proteasomal degradation and activated in response to oxidative stresses. Furthermore, the ROS scavenger N-acetyl cysteine significantly attenuated the anticancer activity of ACA-28. Thus, ACA-28 activates Nrf2 signaling and exerts anticancer activity partly via its ROS-stimulating property. Interestingly, human A549 cancer cells with constitutively high levels of Nrf2 protein showed resistance to ACA-28, as compared with SK-MEL-28. Transient overexpression of Nrf2 also increased the resistance of cells to ACA-28, while knockdown of Nrf2 exerted the opposite effect. Thus, upregulation of Nrf2 signaling protects cancer cells from ACA-28-mediated cell death. Notably, the Nrf2 inhibitor ML385 substantially enhanced the cell death-inducing property of ACA-28 in pancreatic cancer cells, T3M4 and PANC-1. Our data suggest that Nrf2 plays a key role in determining cancer cell susceptibility to ACA-28 and provides a novel strategy for cancer therapy to combine the Nrf2 inhibitor and ACA-28.

## 1. Introduction

The RAS/RAF/MEK/extracellular signal-regulated kinase (ERK) MAPK pathway is a highly conserved signal transduction system that is frequently dysregulated in a large number of human cancers as a result of genetic alterations in their components or upstream activation of cell-surface receptors [1–4]. Aberrant activation of this pathway contributes to tumorigenesis and tumor development [5]. Consequently, tremendous efforts have been dedicated to targeting the ERK signaling pathway for cancer treatment in the past decades, and several inhibitors targeting this pathway have been developed and entering clinical trials [6]. However, the efficacy of targeting RAF/MEK/ERK signaling inhibitors is limited, in part due to acquired resistance caused by gene mutation and bypass activation [7]. Therefore, there is a desperate need to develop innovative therapeutic approaches to fight this deadly malignancy.

Recently, reactive oxygen species (ROS)-inducing therapy, by either accelerating ROS accumulation and/or inhibiting antioxidant processes in cancer cells, has received significant attention for effective anticancer therapy through the selective killing of cancer cells. This can be achieved through the utilization of higher basal ROS levels in cancer cells in comparison with normal cells and the controversial dual roles of ROS in terms of its low-dose contribution to proliferative cell signaling and high-dose cytotoxicity [8, 9]. ROS promote cell proliferation and survival in normal cells. In contrast, excessive levels of ROS above the redox capacity and cytotoxic threshold in cancer cells induced by severe oxidative stress result in selective cancer cell death through the activation of apoptosis or autophagic cell death [10–14]. Therefore, higher endogenous levels of ROS in cancer cells make them more susceptible to prooxidative agents. Indeed, some anticancer drugs currently used in clinics, such as molecular targeted drugs and chemotherapeutic agents, effectively kill cancer cells by inducing ROS generation [13, 15].

Recently, we have identified a novel anticancer compound, ACA-28, through our chemical genetic analysis [16]. We demonstrated that ACA-28 has a unique property to induce ERK-dependent apoptosis in melanoma cell lines (with either BRAF or NRAS mutation) as well as NIH/3T3 cells harboring mutationally active HER2/ErbB2 [16]. ACAGT-007a was obtained from our structure-functional analysis of the original compound ACA-28 [17, 18]. ACAGT-007a was shown to exert a better and more selective anticancer activity than ACA-28 [17]. ACAGT-007a was also shown to be effective in killing pancreatic cancer cell lines with different KRAS mutations in addition to the BRAF-positive SK-MEL-28 melanoma cell lines via ERK-dependent apoptosis [18]. Similar to ACA-28, several anticancer compounds, such as cisplatin, olaparib, and piperlongumine, have been shown to induce cell death by activating ERK signaling [19–21]. Although the repertoire of compounds that mediate ERK activation and apoptosis is expanding [15], a comprehensive understanding of the biological effects induced by ACA-28 remains at a relatively primitive stage.

Here, we have discovered a novel physiological function of ACA-28 to induce ROS. More importantly, ACA-28 exerts anticancer activity partly via its ROS-stimulating property. These findings were derived from our transcriptome analysis in SK-MEL-28 melanoma cells upon exposure to ACA-28 using a DNA microarray, demonstrating that ACA-28 upregulates genes associated with nuclear factor erythroid 2-related factor 2 (Nrf2), a key transcriptional factor that defends oxidative stress [22]. Under homeostatic conditions, Nrf2 is repressed by interaction with a redox-sensitive protein, Keap1 [23]. Keap1 forms part of an E3 ubiquitin ligase, which tightly controls Nrf2 to keep it at a low level by targeting it for ubiquitination and proteasomal degradation in the cytoplasm. In response to oxidative stress, an intricate molecular mechanism facilitated by sensor cysteines within Keap1 allows Nrf2 to escape ubiquitination, and stabilized Nrf2 translocates to the nucleus, thereby activating transcription of various antioxidant and detoxification genes [23]. We further showed that the ACA-28-mediated ROS induction is responsible for the upregulation of the Nrf2 protein.

Intriguingly, although recognized originally as a target of chemopreventive compounds that help prevent cancer and other diseases, accumulating evidence suggests that Nrf2 has a contradictory role in cancers, and overexpression of Nrf2 can promote cancer progression, metastasis, and resistance to anticancer drugs [24]. We, therefore, analyzed the cause–effect relationship between the upregulation of Nrf2 protein and the anticancer activity mediated by ACA-28, based on the controversial dual roles of Nrf2 in cancer. Our data showed that ACA-28-mediated upregulation of Nrf2 signaling protects cancer cells from ACA-28-mediated cell death. Finally, we provide evidence showing that an Nrf2 inhibitor enhanced the ACA-28-mediated cell death in pancreatic cancer, one of the notorious ERK-active cancers. These data may provide novel anticancer strategies to combine the Nrf2 inhibitor and ACA-28, enabling ERK-dependent cancer cells to be more susceptible to ACA-28. Nrf2 may also serve as a determinant for choosing ACA-28-sensitive cancer cell lines.

## 2. Materials and Methods

### 2.1. Reagents

ACA-28 was synthesized as previously described [16] and dissolved in DMSO. The Nrf2 inhibitor ML385 was purchased from Cayman Chemical Company (Ann Arbor, MI, USA) and dissolved in DMSO.

### 2.2. Cell Culture

The human melanoma cell line (SK-MEL-28) was obtained from the Japanese Cancer Research Resources Bank (JCRB) Cell Bank (Osaka, Japan). Human uterine cancer (HeLa), lung cancer (A549), and pancreatic ductal adenocarcinoma (T3M4, PANC-1) cell lines were purchased from the American Type Culture Collection (ATCC) (Manassas, VA, USA). Cells were maintained in Dulbecco's modified Eagle's medium (Nacalai Tesque, Kyoto, Japan) supplemented with 10% fetal bovine serum (BioWest, Nuaillé, Pays de La Loire, France), sodium pyruvate, phenol red, and L-glutamine and were grown at 37˚C in a humidified incubator at 5% CO_2_.

### 2.3. RNA Isolation and Microarray

Total RNA was isolated from SK-MEL-28 cells using RNeasy Mini Kit (Qiagen, Hilden, Germany) according to the manufacturer's instructions. Microarray analysis was performed using Human Transcriptome Array 2.0 (Affymetrix, Santa Clara, CA, USA). According to the Affymetrix recommended protocol, cRNA was prepared from 100 ng total RNA. The cRNA was then used to generate single-stranded DNA, which was fragmented and biotinylated. Labeled single-stranded DNA was hybridized for 16–18 hr at 45°C on Affymetrix HTA 2.0 microarrays, followed by wash and staining with a streptavidin–phycoerythrin conjugate in an Affymetrix Fluidics Station 450. Genechips were scanned with a GeneChip Scanner 3000 7G (Affymetrix) according to the manufacturer's guidelines. The CEL files generated were analyzed through Affymetrix Expression Console Software, which normalizes the array signals using a robust multiarray averaging algorithm. Normalized data were analyzed using Transcriptome Analysis Console Software (Affymetrix).

### 2.4. Protein Extraction and Western Blot Analysis

The mammalian cell lines were treated with chemicals, as described in the legend of each figure. The harvested cells were lysed and subjected to immunoblotting as previously described [16]. The following primary antibodies were used: anti-Nrf2 (EP1808Y) rabbit mAb (Abcam, #ab62352) and anti-GAPDH (14C10) Rabbit mAb (Cell Signaling Technology, Danvers, MA, USA, #2118). As a secondary antibody, an anti-rabbit (#7074) IgG HRP-linked antibody (Cell Signaling Technology) was used. The proteins were detected by Chemi–Lumi One Super (Nacalai Tesque) or ECL Select (Cytiva, Marlborough, MA, USA). Relative intensities of all bands were quantified using MULTI GAUGE Ver. 3.2 software (Fujifilm, Tokyo, Japan).

### 2.5. ROS Measurement

ROS generation was determined using a CellROX™ Deep Red Reagent (Thermo Fisher Scientific, Waltham, MA, USA). The fluorescence intensity of CellROX Deep Red reflects the ROS levels. Briefly, cells were seeded in a 12-well plate and incubated for 24 hr. After 1 hr of preincubation with N-acetyl cysteine (NAC) (5 mM) or mock, the CellROX Deep Red reagent was added to the samples at a final concentration of 5 *µ*M and incubated for 15 min at 37˚C in the dark, then ACA-28 or TBHP was added to the samples at a final concentration of 20 and 50 *µ*M, respectively, and incubated for another 60 min at 37˚C in the dark. The medium was then removed, and the cells were harvested with trypsin-EDTA (Sigma) and washed two times with PBS. The cells were then subjected to flow cytometry using a BD LSRFortessa™ flow cytometer (BD Biosciences, Franklin Lakes, NJ, USA) (excitation/emission wavelengths: 640/670 nm) and analyzed by FlowJo version 10.

### 2.6. Cell Viability Assay

Cell viability was measured using a Cell Count Reagent SF (Nacalai Tesque Inc.) according to the manufacturer's instructions with small modifications. Briefly, 100 *µ*l of mammalian cell suspension was seeded at a cell density of 5.0 × 10^4^ cells/ml in a 96-well plate (IWAKI, Shizuoka, Japan) and incubated for 24 hr. In each medium, the compounds in solution were diluted 1 : 1,000, and 100 *µ*l of the diluted compounds were added to the cell culture. Cells treated with solvent (DMSO) were used as controls. After incubation for 48 hr, a mixture of 5 *µ*l of Cell Count Reagent SF and 45 *µ*l of the medium was added to each well, followed by incubation for an additional 3 hr. Then, the absorbance at 450 nm was measured using a Sunrise microplate reader (Tecan, Männedorf, Switzerland). Absorbance at 600 nm was also measured as a reference.

### 2.7. Knockdown and Overexpression of Nrf2

A pool of three target-specific Stealth siRNAs targeting human Nrf2/NFE2L2 (HSS107130, HSS181505, and HSS181506) and a control siRNA (Stealth RNAi™ Negative Control Med GC Duplex) were purchased from Invitrogen USA. The pcDNA™5-FLAG-HA-SBP-Nrf2 expressing plasmid (pKD4354) and its control vector was a kind gift from Dr. Akio Yamashita. Eighty microliters of 4 × 10^4^ cells/ml were seeded in a 96-well plate (IWAKI) and incubated for 24 hr. The cells were transfected with 10 nM siRNA using a Lipofectamine RNAiMAX transfection reagent (Thermo Fisher Scientific) at a concentration of 0.91 *μ*l/well or with 0.1 ng/*µ*l of the Nrf2 expressing plasmid using a PEI MAX transfection reagent (final medium volume 100 *µ*l/well), and incubated for 24 hr. Fifty microliters of the diluted ACA-28 were added to the cell culture and incubated for 24 hr, then subjected to the cell viability assay as described above.

### 2.8. Statistical Analysis

The significance of the data was evaluated as described in the legend of each figure.

## 3. Results

### 3.1. ACA-28 Induces the Expression of Genes Associated with Nrf2

**Figure 1:**
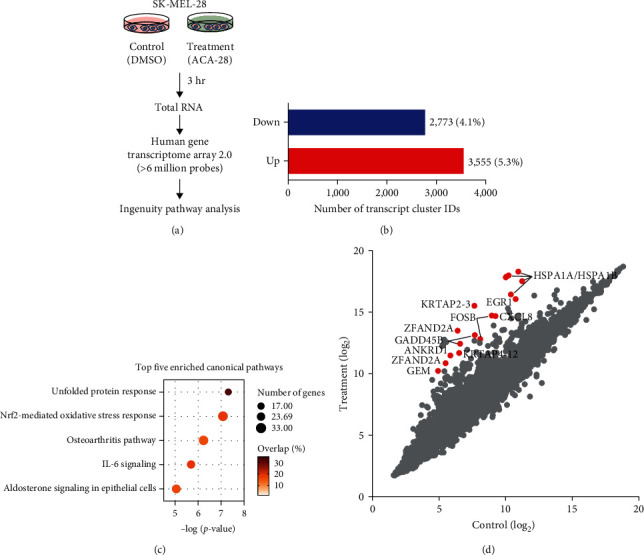
ACA-28 induces the expression of genes associated with Nrf2. (a) Workflow for the identification of genetic pathways altered by ACA-28. (b) Bar graph showing the number of Transcript Cluster IDs (TC IDs) upregulated (>1.5 fold) and downregulated (<−1.5 fold) by ACA-28, among the 67528 IDs identified by human transcriptome array analysis. (c) Bubble plot representing the top five canonical pathways enriched by ACA-28, identified by Ingenuity pathway analysis. Bubble size, the number of genes assigned to a pathway; color, the overlapping percentage for genes belonging to the indicated pathways. (d) Scatter plot showing the expression level of TC IDs with annotated gene names. The dots indicate the intensity levels of each TC ID. Representative TC IDs corresponding to the top 11 genes upregulated by ACA-28 are shown in red. The 11 genes contain consensus binding motifs for Nrf2, as shown in Figure S1.

To comprehensively understand the biological effects elicited by ACA-28, we performed a global gene expression analysis using a DNA microarray (Human Transcriptome Array 2.0). SK-MEL-28 melanoma cells were treated with ACA-28 or DMSO, and the differentially expressed genes were screened from the gene expression profile between the two samples treated with or without ACA-28 (Figure 1(a)).

The ACA-28-treated cells had a total of 6,328 Transcript Cluster IDs (TC IDs) that showed a change of more than 1.5-fold increase or decrease compared with those in the cells treated with vehicle, in which 3,555 were upregulated, and 2,773 were downregulated, indicating that ACA-28 leads to significant changes in gene expression (Figure 1(b)). Using Ingenuity pathway analysis (IPA) for genes corresponding to these TC IDs, five canonical pathways were found to be enriched (Figure 1(c)). These pathways include the unfolded protein response, the Nrf2-mediated oxidative stress response, the osteoarthritis pathway, the IL-6 signaling, and the aldosterone signaling. The top five negatively enriched pathways are also shown in Figure S1(a). Importantly, the top 11 genes upregulated by ACA-28 (Figure 1(d), indicated by red dots) contain the core Nrf2 consensus binding motifs within −1 kb of their transcription start sites (TSS), as shown in Figure S1(b).

**Figure 2:**
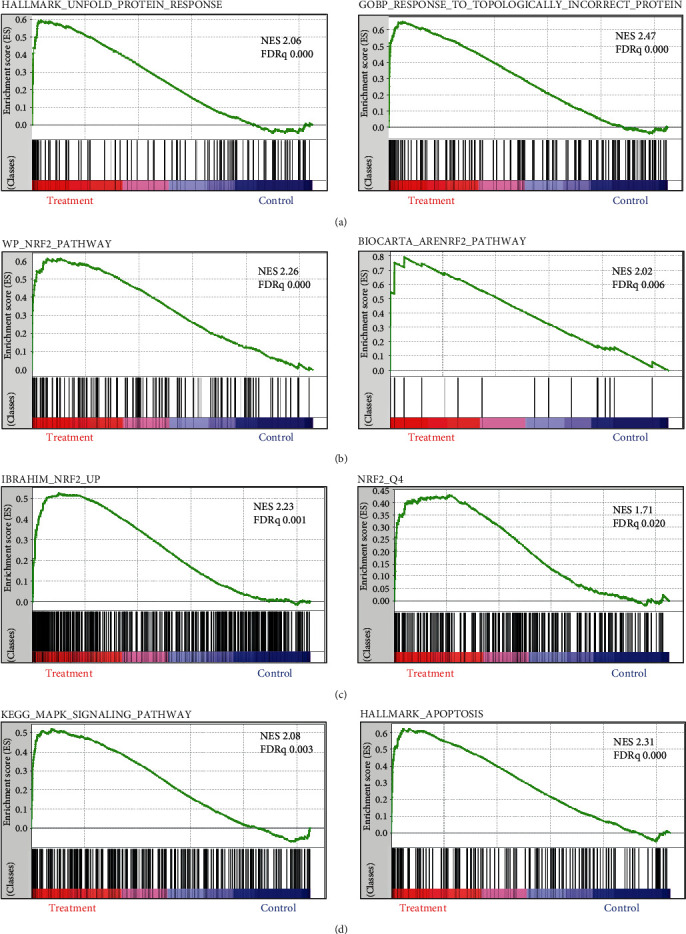
ACA-28 comprehensively upregulates the expression of genes in selected pathways. (a and b) GSEA plots showing a significant increase in genes associated with unfolded protein response (a) and genes associated with the Nrf2 pathway (b) in ACA-28-treated cells. (c) GSEA plots showing a significant increase in genes induced by Nrf2 overexpression (IBRAHIM_Nrf2_UP) and genes having the Nrf2 motif in the regions spanning 4 kb centered on their transcription starting sites (−2 kb, +2 kb) (Nrf2_Q4) in the treatment group. (d) GSEA plots showing a significant increase in genes associated with MAPK signaling (KEGG_MAPK_SIGNALING_PATHWAY) and apoptosis (HALLMARK_APOPTOSIS) in the treatment group. Treatment, cells treated with ACA-28; Control, cells treated with DMSO; NES, normalized enrichment score; FDRq, false discovery rate *q*-value.

To further confirm the results of IPA, gene set enrichment analysis (GSEA) was performed. The results showed that the genes related to unfolded protein response (Figure 2(a)) and the genes related to the Nrf2 pathway (Figure 2(b)) were significantly increased in the ACA-28-treated samples. In addition, genes induced by Nrf2 overexpression (Figure 2(c), IBRAHIM_Nrf2_UP) and genes that have the Nrf2 motif in the regions spanning 4 kb centered on their TSS (−2 kb, +2 kb) (Figure 2(c), Nrf2_Q4) were increased in the treatment group. Furthermore, genes associated with MAPK signaling (Figure 2(d), KEGG_MAPK_SIGNALING_PATHWAY) and apoptosis (Figure 2(d), HALLMARK_APOPTOSIS) were enriched in the treatment group, supporting our previous findings that ACA-28 induces ERK-dependent apoptosis. These findings prompted us to elucidate the functional relationship between ACA-28 and Nrf2 signaling.

### 3.2. ACA-28 Activates Nrf2 Signaling by Increasing Nrf2 Protein Levels via ROS Production

**Figure 3:**
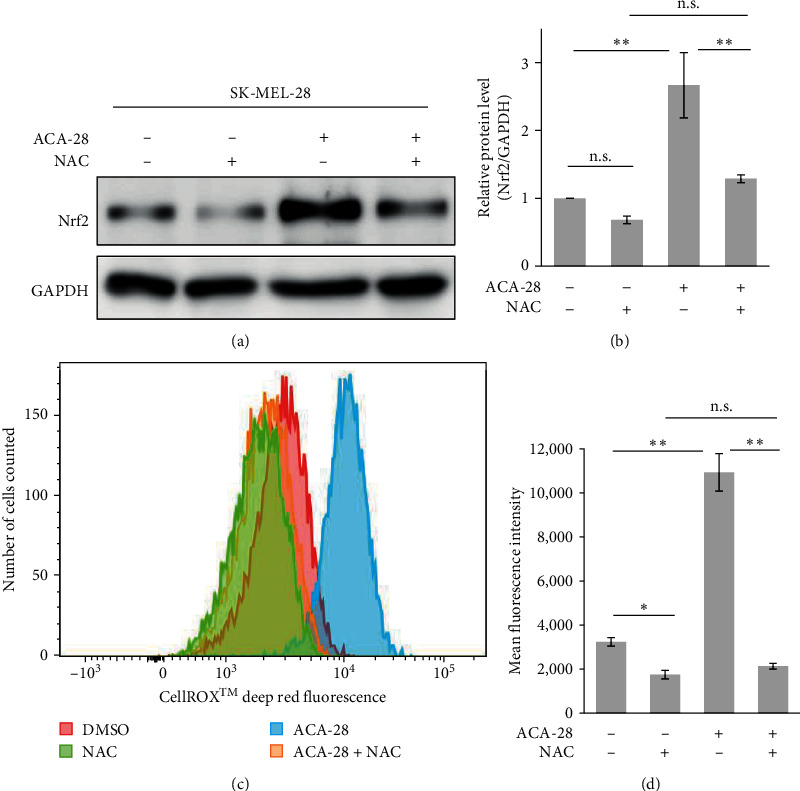
ACA-28 increases ROS and Nrf2 protein levels in SK-MEL-28. (a) A representative Western blot analyzing the Nrf2 and GAPDH expression in SK-MEL-28 cells treated with ACA-28 (10 *µ*M) or DMSO for 6 hr, with or without pretreatment with NAC (5 mM) for 1 hr. GAPDH was used as an internal control. (b) Relative quantification of Nrf2 protein levels shown in (a). Data are expressed as the mean ± standard deviation (SD) from three independent experiments. (c) Representative histogram of ROS levels in SK-MEL-28 cells assessed by fluorescence intensity of CellROX Deep Red using flow cytometry. Cells were treated with ACA-28 (20 *µ*M) or DMSO for 1 hr, with or without pretreatment with NAC (5 mM) for 45 min. (d) The mean fluorescence intensity (MFI) of the data is shown in (c). Data are presented as the mean ± standard deviation (SD) from three independent experiments (n.s.: not significant,  ^*∗*^*p* < 0.05,  ^*∗∗*^*p* < 0.01, Tukey's multiple comparison test).

Under oxidative stress, an increased level of intracellular ROS has been shown to promote the dissociation of Nrf2 and Keap1, which stabilizes Nrf2, thereby activating the transcription of various antioxidant and detoxification genes [25, 26]. As a first step toward understanding the mechanism of ACA-28-mediated Nrf2 signaling activation, we tried to investigate whether ACA-28 treatment affects Nrf2 protein levels. Nrf2 protein was detected using anti-Nrf2 antibodies in SK-MEL-28 cells. Upon ACA-28 treatment, significant upregulation of the Nrf2 protein was observed in SK-MEL-28 cells (approximately threefold) (Figures 3(a) and 3(b)). Importantly, pretreatment with the antioxidant NAC significantly reduced the Nrf2 protein levels in SK-MEL-28 cells with or without ACA-28 treatment.

Next, we investigated whether ACA-28 can induce ROS. To evaluate ROS levels, we used the flow cytometric method with the CellROX Deep Red Reagent (see Section 2). SK-MEL-28 melanoma cell lines were incubated with or without 20 *μ*M ACA-28 for 30 min. Flow cytometric analysis showed that SK-MEL-28 cells treated with ACA-28 reached higher intensity staining with CellROX Red reagent compared to those treated with vehicle (DMSO) (Figures 3(c) and 3(d). TBHP (tert-butyl hydroperoxide) was used as a control oxidant that stimulates ROS. SK-MEL-28 cells treated with 50 *µ*M of TBHP have significantly increased staining with the reagent comparable to those treated with ACA-28 (Figure S2). Importantly, pretreatment with NAC for 15 min markedly reduced the fluorescence in the cells treated with ACA-28 (Figures 3(c) and 3(d)), confirming that the increase in the CellROX fluorescence induced by ACA-28 is mediated by ROS.

### 3.3. ACA-28 Exerts Anticancer Activity Partly via Its ROS-Stimulating Property

**Figure 4:**
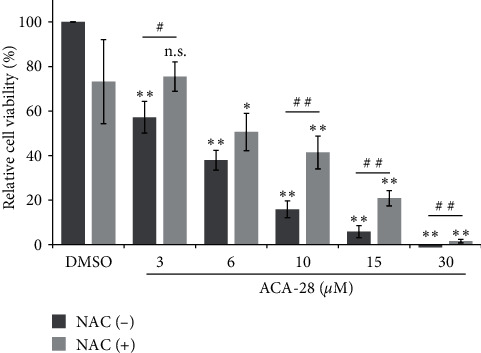
NAC attenuates the loss of cell viability induced by ACA-28. Viability of SK-MEL-28 cells after exposure to the indicated concentrations of ACA-28 with or without 2 mM NAC for 48 hr. Cell viability was assessed with the WST-8 assay. Data are expressed as the relative percentage to that of DMSO treatment. Bars represent the mean ± SD of three independent experiments (n.s.: not significant,  ^*∗*^*p* < 0.025,  ^*∗∗*^*p* < 0.005, Williams' post hoc test compared to the respective DMSO control) (^#^*p*  < 0.05, ^##^*p*  < 0.01, unpaired Student's *t*-test between two groups).

We next investigated if the ROS production by ACA-28 is functionally associated with its anticancer activity. We then treated SK-MEL-28 cells with various concentrations of ACA-28 in the presence or absence of NAC, and cell viability was analyzed. As shown in Figure 4, ACA-28 significantly reduced the cell viability in a dose-dependent manner, as reported in previous studies [16, 17], and approximately less than 20% of cells are viable when cells are treated with ACA-28 at concentrations higher than 10 *μ*M. Simultaneous treatment of SK-MEL-28 cells with NAC significantly recovered the loss of cell viability by ACA-28 (Figure 4).

### 3.4. Human Non-Small Lung Cancer A549 Cells, Which Possess Constitutively Active Nrf2, Showed Resistance to ACA-28

**Figure 5:**
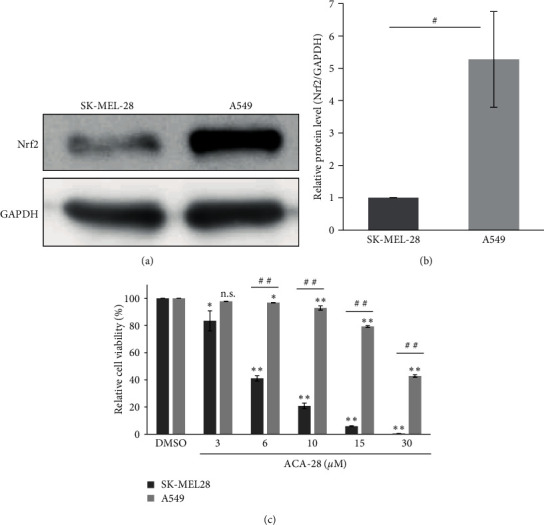
A549, a cell line with constitutively activated Nrf2, is resistant to ACA-28. (a) A representative western blot showing the expression of Nrf2 and GAPDH in SK-MEL-28 and A549 cells. (b) Relative quantification of the Nrf2 protein levels shown in (a). GAPDH was used as an internal control. Data are expressed as the mean ± standard deviation (SD) from three independent experiments. (c) Comparison of the viability of SK-MEL-28 and A549 cells after exposure to the indicated concentrations of ACA-28 for 48 hr. Cell viability was assessed with the WST-8 assay. Data are expressed as the relative percentage to that of DMSO treatment. Bars represent the mean ± standard deviation (SD) from two independent experiments (n.s.: not significant,  ^*∗*^*p* < 0.025,  ^*∗∗*^*p* < 0.005, Williams' post hoc test compared to the respective DMSO control) (^#^*p*  < 0.05, ^##^*p*  < 0.01, unpaired Student's *t*-test between two groups).

We next wanted to investigate whether ACA-28-mediated upregulation of Nrf2 endows cancer cells with resistance to ACA-28 or whether upregulation of Nrf2 is functionally relevant to the loss of cell viability by ACA-28. Nrf2 has been reported to be upregulated in some cancer cells, as represented in human lung cancer A549 cells [27, 28]. Consistently, the expression levels of Nrf2 in A549 cells were significantly higher (approximately fivefold) than those in SK-MEL-28 cells (Figures 5(a) and 5(b)). We then compared the cell viability of these two strains upon ACA-28 treatment (Figure 5(c)). Importantly, the resistance to ACA-28 was opposite to Nrf2 expression, with the IC_50_ values of ACA-28 in A549 cells and SK-MEL-28 cells being 27.9 and 5.2 *μ*M, respectively.

### 3.5. The Overexpression and Silencing of Nrf2 Affect the ACA-28 Sensitivity in HeLa Cells

**Figure 6:**
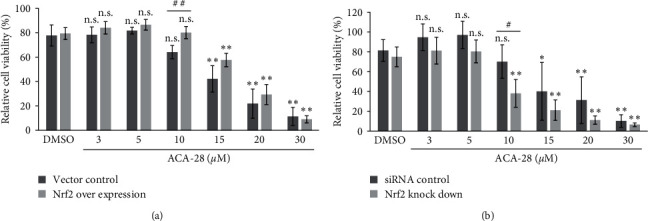
Manipulation of Nrf2 protein levels affects the ACA-28 sensitivity in HeLa cells. (a) HeLa cells were transfected with pcDNA5-Nrf2-HA or the control vector and subsequently treated with the indicated concentrations of ACA-28 for 24 hr. (b) HeLa cells were transfected with scrambled siRNA or Nrf2 siRNA and subsequently treated with the indicated concentrations of ACA-28 for 24 hr. Cell viability was assessed with the WST-8 assay. Data are expressed as the relative percentage to that of nontransfected cells treated with DMSO (0 *µ*M of ACA-28). Bars represent the mean ± SD of four independent experiments (n.s.: not significant,  ^*∗*^*p* < 0.025,  ^*∗∗*^*p* < 0.005, Williams' post hoc test compared to the respective DMSO control) (^#^*p*  < 0.05, ^##^*p*  < 0.01, unpaired Student's *t*-test between two groups).

To directly ask if the expression levels of Nrf2 play a key role in determining the cancer cells' sensitivity to ACA-28, we try to manipulate the Nrf2 expression in two ways. First, we overexpressed the Nrf2 gene in HeLa cells (Figure 6(a)). HeLa cells express a slightly higher level of Nrf2 protein as compared with SK-MEL-28 cells (Figure S3). Cell viability of HeLa cells overexpressing the Nrf2 gene was significantly higher compared to that of cells harboring the control vector alone when treated with 10 *μ*M ACA-28 (Figure 6(a)). Second, we investigated the effect of Nrf2 knock-down on HeLa cells' susceptibility to ACA-28 (Figure 6(b)). Nrf2 expression was downregulated by Nrf2 siRNA. The effect of Nrf2 silencing on Nrf2 protein levels compared to control siRNA was confirmed by immunoblotting (Figure S4). Nrf2 silencing sensitizes HeLa cells to ACA-28, as evaluated by the WST-8 assay, as cell viability was significantly reduced when cells were treated with Nrf2 siRNA and 10 *μ*M ACA-28 (Figure 6(b)).

### 3.6. Nrf2 Inhibitor Increases the Sensitivity to ACA-28 in Pancreatic Cancer Cell Lines

**Figure 7:**
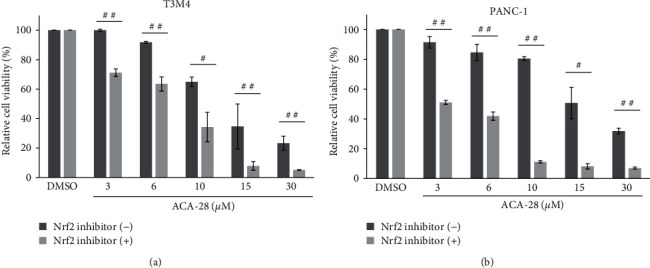
Nrf2 inhibitor increases the sensitivity to ACA-28 in the pancreatic cancer cell lines, T3M4 and PANC-1. (a, and b) Comparison of the viability of T3M4 (a) and PANC-1 cells (b) after exposure to the indicated concentrations of ACA-28 with or without 10 *µ*M of the Nrf2 inhibitor, ML385, for 48 hr. Cell viability was assessed with the WST-8 assay. Data are expressed as the relative percentage to that of DMSO treatment. Bars represent the mean ± SD of three independent experiments (n.s.: not significant,  ^*∗*^*p* < 0.025,  ^*∗∗*^*p* < 0.005, Williams' post hoc test compared to the respective DMSO control) (^#^*p*  < 0.05, ^##^*p*  < 0.01, unpaired Student's *t*-test between two groups).

To facilitate Nrf2 inhibition by a simple method, we next examined the effect of an Nrf2 inhibitor in combination with ACA-28 on cancer cell viability. For this, we explored the activity of a small molecule Nrf2 inhibitor ML385, which inhibits the DNA-binding activity of the Nrf2-MAFG [29], on the viability of PDAC cell lines with ACA-28. ACA-28 inhibited the growth of two PDAC cell lines, T3M4 and PANC-1 (Figure 7). The addition of ML385 further exacerbated the loss of cell viability induced by ACA-28 in both cell lines (Figure 7).

## 4. Discussion

ROS have dual roles in oncogenesis. ROS can promote prooncogenic signaling, facilitating cancer cell proliferation, survival, and adaptation to hypoxia. Moreover, cancer cells exhibit higher basal levels of ROS compared with normal cells as a result of an imbalance between oxidants and antioxidants. On the other hand, ROS can promote antioncogenic signaling and trigger oxidative stress-induced cancer cell death [14, 30, 31]. Here, we provide evidence that ACA-28, an anticancer compound, induces cancer cell death partly via its ROS-stimulating properties, inspired by our transcriptome analysis showing that Nrf2, a master transcription factor of neutralizing cellular ROS, is upregulated by ACA-28. Importantly, the upregulation of Nrf2 turns out to protect cancer cells from ACA-28-mediated cell death.

### 4.1. ROS Induction and ERK-Dependent Apoptosis Mediated by ACA-28

A unique feature of ACA-28 is its ability to stimulate ERK-dependent apoptosis in several cancer cell lines, including SK-MEL-28 melanoma cells [16]. ACA-28 and its lead compound ACAGT-007a were shown to induce apoptosis in cancer cell lines with different oncogenic mutations, ranging from HER2, BRAF, NRAS, and KRAS [16, 18]. Although the precise underlying mechanisms of ERK-dependent cell death remain largely unclear, one of the properties shared by several compounds capable of inducing ERK activation-dependent apoptosis is ROS stimulation [15]. ROS can attack the cysteine residues of a certain group of target proteins, including MAP kinase phosphatases/dual-specific phosphatases (DUSPs), thereby oxidizing the reactive thiol groups to form a disulfide bond, which leads to inactivation of DUSPs and stimulation of ERK activity [32]. Importantly, ACA-28 and ACAGT-007a have a unique structure similar to ACA, which can be converted to a quinone methide intermediate by eliminating the two carbonate esters [17, 33]. The quinone methide intermediate can be converted to ROS-like substances (e.g., peroxide) by reacting with oxygen. Therefore, the stimulation of the ROS-like substance derived from ACA-28 and ACAGT-007a is attenuated by the ROS scavenger NAC. Consistently, our data showed that ACA-28 stimulates cellular ROS levels to a similar extent as achieved by a strong oxidant TBHP (Figure S2).

Our transcriptome analysis leads to the discovery that Nrf2 signaling is an important downstream pathway that is activated by ACA-28 via its ROS-inducing properties. Notably, the Keap1/Nrf2 axis is a well-established defense mechanism to combat oxidative stress, and Nrf2 can be activated by ROS through the inactivation of its cytoplasmic repressor protein, Keap1 [34]. Human Keap1 is a thiol-rich protein harboring 27 cysteine residues, which are targets of oxidation by ROS [35]. This modification on Cys residues leads to conformational changes in Keap1, disrupting the Keap1/Nrf2 interaction, thus inhibiting the polyubiquitination of Nrf2, enabling its escape from proteasomal degradation [34]. Based on the shared structure with ACA, ACA-28, via the formation of the quinone methide intermediate, it may serve as an oxidant to attack the cysteine residues of the target proteins, including Keap1. The findings that NAC canceled both the upregulation of Nrf2 protein levels and anticancer activity mediated by ACA-28 suggest that ROS induction is an important mechanism of cell death and Nrf2 activation by ACA-28. It should be noted that although NAC almost perfectly attenuated ROS stimulation and Nrf2 upregulation by ACA-28, the recovery of cell viability by NAC was not full (Figures 3 and 4), suggesting that ACA-28 induces cell death via multiple mechanisms.

Other mechanisms that might be involved in inducing apoptosis by ACA-28 may include p53 and mitochondria. DNA-damaging stimuli are one of the largest mechanisms associated with ERK-dependent apoptosis. For example, several DNA damage-inducing drugs, such as etoposide and cisplatin, or UV treatment can induce ERK-dependent apoptosis in various cancer cell lines [15, 36–38]. DNA damage can activate p53 by inducing a rapid increase in p53 protein levels and a subsequent increase in its transcriptional activity, which results in the induction of several genes whose products trigger cell-cycle arrest, DNA repair, or apoptosis [39]. Thus, it would be intriguing to speculate that ACA-28 may serve as a DNA-damaging agent, thereby inducing apoptosis via p53 activation. Another possible mechanism related to ERK-dependent apoptosis involves mitochondrial activation. Several studies suggested that ERK may act on mitochondria to induce cytochrome *c* release through a pro-apoptotic molecule Bax and/or p53 [40–42]. For example, cisplatin-induced apoptosis is mediated by ERK activation to induce mitochondria membrane depolarization and cytochrome *c* release, and caspase-3 activation [41]. Importantly, cisplatin-induced expression of Bax and p53 was decreased by the ERK pathway inhibition [42]. Whether ACA-28 can induce p53 by serving as a DNA damage-inducing agent and/or whether ACA-28-mediated ERK activation can induce cytochrome c release and caspase-3 activation via mitochondria will be important issues for future study.

### 4.2. Role of Nrf2 Signaling in Cancer Cell Death Mediated by ACA-28

An additional important aspect of our findings is the functional relevance of ACA-28-mediated upregulation of Nrf2 signaling and its properties to kill cancer cells. Nrf2 has a dual role in cancer progression. If Nrf2 protects cells from oxidative stress elicited by ACA-28, upregulation of Nrf2 will endow cancer cells with resistance to ACA-28. In contrast, if ACA-28 induces cancer cell death by inducing Nrf2, upregulation of Nrf2 may exert opposite effects on cancer cell growth. To investigate the relationship between cellular Nrf2 expression levels and resistance to ACA-28, we used the A549 human lung cancer cell line, which harbors the Keap1 mutation expressing high Nrf2 protein levels [27]. The human A549 cells showed significantly higher resistance to ACA-28 as compared with SK-MEL-28 cells expressing relatively low levels of Nrf2 (Figure 5(c)). The overexpression and silencing experiments of Nrf2 expression further support our conclusion that upregulation of Nrf2 signaling protects cancer cells from ACA-28-mediated cell death. How does the upregulation of Nrf2 protect cancer cells from various anticancer agents? Nrf2 regulates the gene expression of a number of detoxifying enzymes, including *γ*-glutamylcysteine synthetase, via the antioxidant response element (ARE), thereby increasing cellular antioxidants as represented by glutathione [43]. This property of Nrf2 overexpression has been reported to endow resistance to various anticancer compounds ranging from chemotherapeutic agents such as cisplatin to molecularly targeted drugs, as exemplified by imatinib [44, 45]. Importantly, a reducing agent, such as ascorbic acid, can overcome the resistance to imatinib, consistent with the idea that changes in the redox state caused by antioxidants can affect imatinib resistance via inhibition of Nrf2-mediated gene expression [46]. In the case of human lung carcinoma A549 cells, which express constitutively active Nrf2, the flavonoid luteolin induced a marked reduction in Nrf2 levels, thereby downregulating ARE-driven genes and depletion of reduced glutathione [47].

Finally, our findings using the Nrf2 inhibitor ML385 also support the above conclusion and suggest an attractive combination therapy of ACA-28 with an Nrf2 inhibitor to effectively inhibit cancer cell viability. Since Nrf2 signaling is aberrantly activated in several cancers such as skin, lung, ovarian, breast, bladder, and pancreatic cancers [48]. Thus, the diagnosis of Nrf2 activation could facilitate the use of Nrf2 inhibitors in combination with ACA-28.

## 5. Conclusions

ACA-28 is effective in killing ERK-active cancer cells as represented by melanoma and pancreatic cancers. ACA-28 was shown to exert anticancer activity partly via its ROS-stimulating property. ACA-28-mediated Nrf2 upregulation protects cancer cells from cell death. Additionally, the combined treatment with ACA-28 and an Nrf2 inhibitor effectively enhanced the cell death-inducing property of ACA-28, thus providing a possible therapeutic approach for some ERK-active cancer cells.

## Data Availability

The datasets generated during the current study are available from the corresponding authors upon reasonable request.

## Supplementary Materials

Supplementary Materials Figure S1: supplementary results corresponding to Figure 1. (a) Top five negatively enriched pathways in cells treated with ACA-28 identified by Ingenuity Pathway Analysis. (b) The consensus binding motifs for Nrf2 are seen in the top genes upregulated by ACA-28 treatment. Figure S2: comparison of ROS levels in cells treated with TBHP and ACA-28. SK-MEL-28 cells were treated with DMSO (blue), 50 *µ*M of TBHP (green), or 20 *µ*M of ACA-28 (red) for 1 hr. Representative histograms and the mean fluorescence intensity (MFI) were shown. Bar graph is presented as the mean ± standard deviation (SD) of three independent experiments. Figure S3: comparisons of the Nrf2 protein levels in SK-MEL-28, A549, and HeLa cells. (a) Representative Western blot analyzing the Nrf2 and GAPDH expression in SK-MEL-28, A549, and HeLa cells. (b) Relative quantification of the Nrf2 protein levels shown in (a). GAPDH was used as an internal control. Data are expressed as the mean ± standard deviation (SD) from three independent experiments. Figure S4: Nrf2 protein levels in siRNA treated HeLa cells. (a) Western blot analyzing the Nrf2 and GAPDH expression in control siRNA or Nrf2 siRNA -treated HeLa cells. (b) Relative quantification of the Nrf2 protein levels shown in (a). GAPDH was used as an internal control.
