# Supplementary material for: ACA-28, an ERK MAPK Signaling Modulator, Exerts Anticancer Activity through ROS Induction in Melanoma and Pancreatic Cancer Cells
Source: Oxid Med Cell Longev. 2024 Mar 11;2024:7683793. doi: 10.1155/2024/7683793 (PMC10948229; doi:10.1155/2024/7683793)

Supplemental Information

ACA-28, an ERK MAPK signaling modulator, exerts anticancer activity through ROS induction in melanoma and pancreatic cancer cells.

Teruaki Takasaki,[1] Yasuyuki Hamabe,[1] Kenta Touchi,[1] Golam Iftakhar Khandakar,[1] Takeshi Ueda,[2,5], Hitoshi Okada,[2,5] Kazuko Sakai,[3] Kazuto Nishio,[3] Genzo Tanabe,[4] and Reiko Sugiura[1,5]

[1] Laboratory of Molecular Pharmacogenomics, Department of Pharmaceutical Sciences, Faculty of Pharmacy, Kindai University, Osaka 577-8502, Japan
[2] Department of Biochemistry, Kindai University Faculty of Medicine, Osaka 589-8511, Japan
[3] Department of Genome Biology, Kindai University Faculty of Medicine, Osaka 589-8511, Japan
[4] Laboratory of Organic Chemistry, Department of Pharmacy, Faculty of Pharmacy, Kindai University, Osaka 577-8502, Japan
[5] Anti-Aging Center, Kindai University, Osaka 577-8502, Japan

*To whom correspondence should be addressed:
TEL: +81-6-4307-3642; FAX: +81-6-6730-1394, E-mail: sugiurar@phar.kindai.ac.jp

Supplementary Figure 1

a

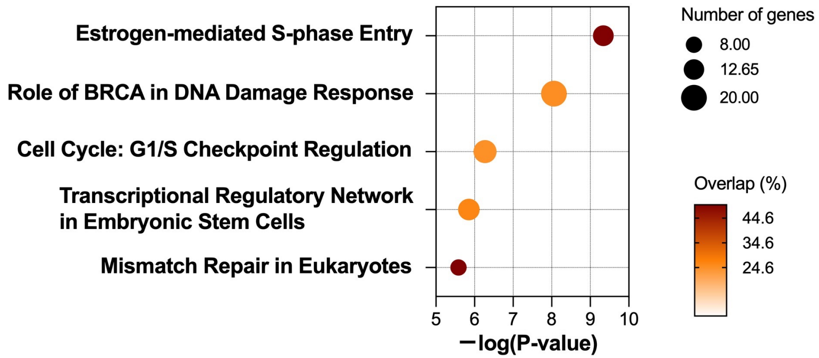

b

Gene symbol	Description	Binding consensus for NRF2
KRTAP2-3 /KRTAP2-4	keratin associated protein	TGCCTCAGCA-118 TGCCTCAGCA-128
HSPA1A /HSPA1B	heat shock 70kDa protein	TGGCTCAGAA-58 TGGCTCAGAA-57
ZFAND2A	zinc finger AN1-type containing 2A	TGACCCAGCA-203
GADD45B	growth arrest and DNA damage inducible beta	TGACTTAACA-495
FOSB	AP-1 transcription factor subunit	TGTCTCCGGG-917
ANKRD1	ankyrin repeat domain 1	TGTCTCTGCC-653
CXCL8	C-X-C motif chemokine ligand 8	TGACTCAGGT-130
EGR1	early growth response 1	TGGCCCGGCC-334
GEM	GTP binding protein overexpressed in skeletal muscle	AGCCTCAGCA-681 TGCCACTGCA-440
KRTAP4-12	keratin associated protein 4-12	GTTCTCAGCA-34

Supplementary Figure 2

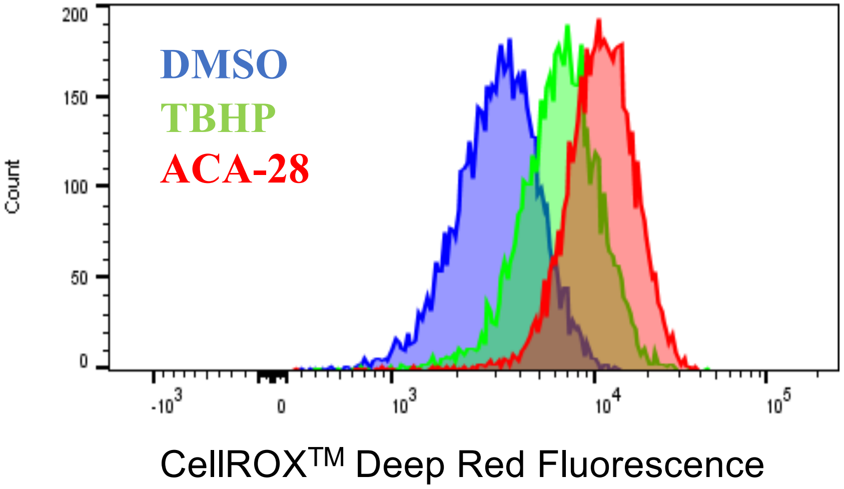

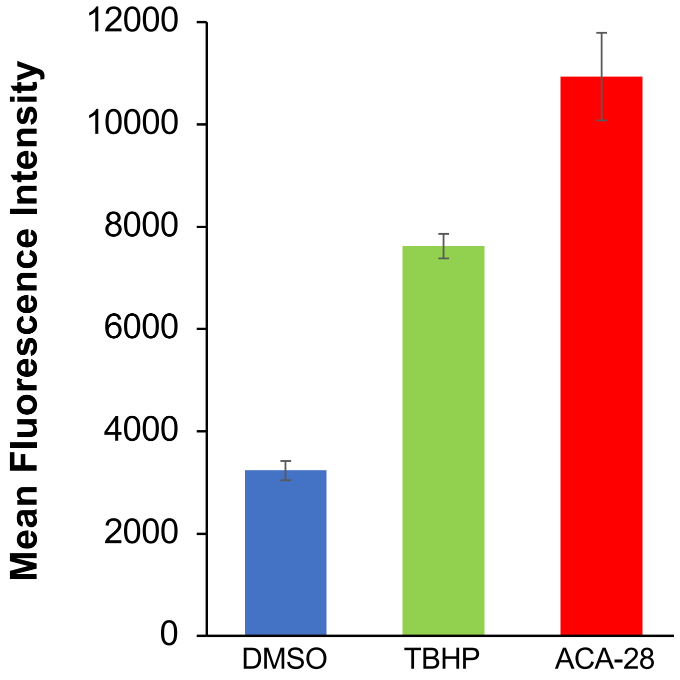

Supplementary Figure 3

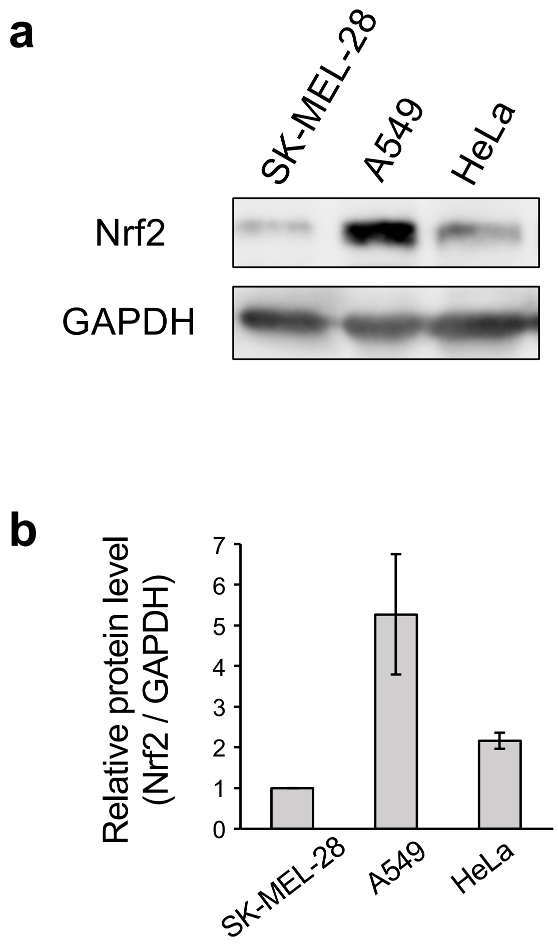

Supplementary Figure 4

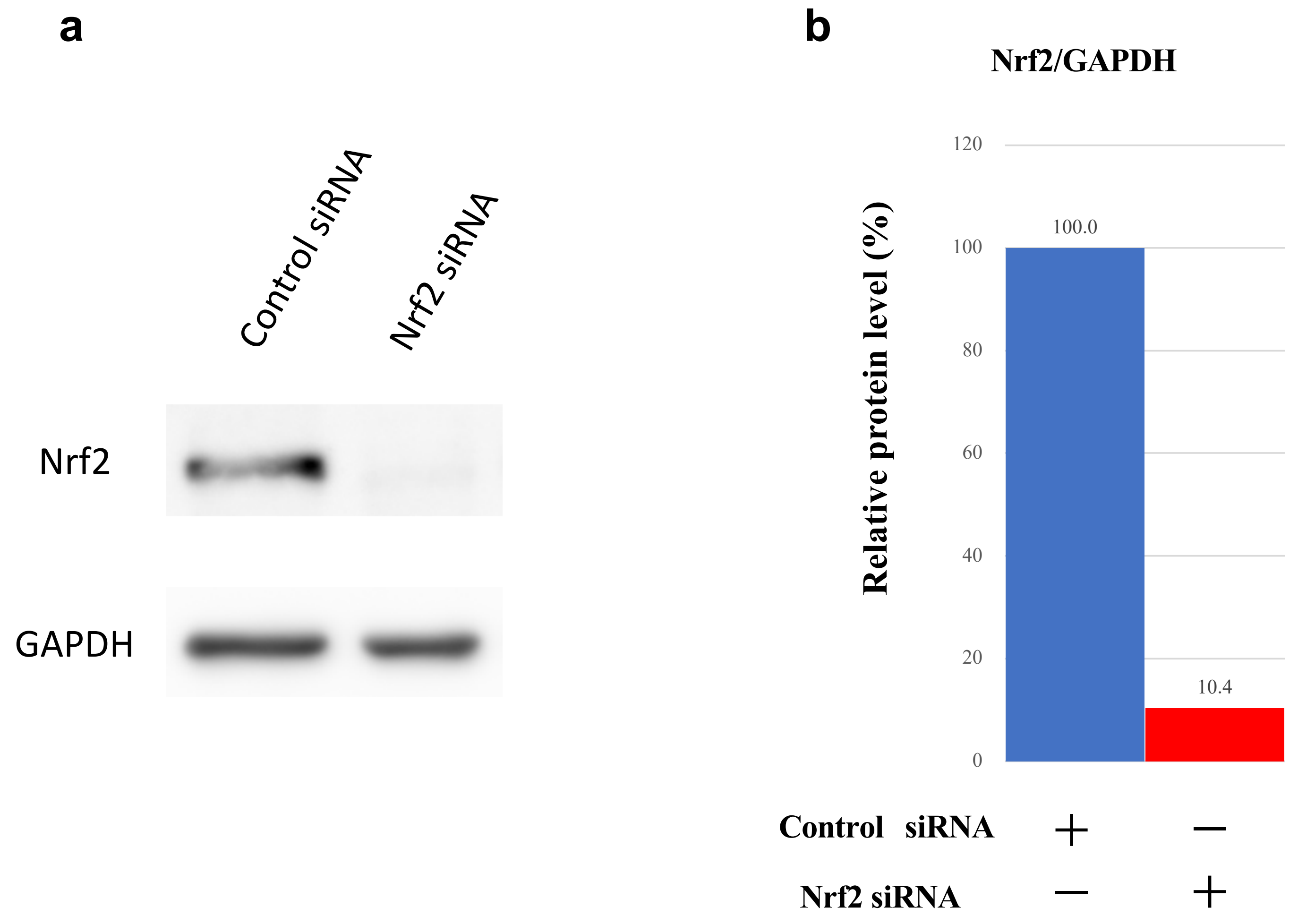

Supplement: Supplementary Materials — Figure S1: supplementary results corresponding to Figure 1. (a) Top five negatively enriched pathways in cells treated with ACA-28 identified by Ingenuity Pathway Analysis. (b) The consensus binding motifs for Nrf2 are seen in the top genes upregulated by ACA-28 treatment. Figure S2: comparison of ROS levels in cells treated with TBHP and ACA-28. SK-MEL-28 cells were treated with DMSO (blue), 50 µM of TBHP (green), or 20 µM of ACA-28 (red) for 1 hr. Representative histograms and the mean fluorescence intensity (MFI) were shown. Bar graph is presented as the mean ± standard deviation (SD) of three independent experiments. Figure S3: comparisons of the Nrf2 protein levels in SK-MEL-28, A549, and HeLa cells. (a) Representative Western blot analyzing the Nrf2 and GAPDH expression in SK-MEL-28, A549, and HeLa cells. (b) Relative quantification of the Nrf2 protein levels shown in (a). GAPDH was used as an internal control. Data are expressed as the mean ± standard deviation (SD) from three independent experiments. Figure S4: Nrf2 protein levels in siRNA treated HeLa cells. (a) Western blot analyzing the Nrf2 and GAPDH expression in control siRNA or Nrf2 siRNA -treated HeLa cells. (b) Relative quantification of the Nrf2 protein levels shown in (a). GAPDH was used as an internal control. [file 7683793.f1.pdf]
